# Perception and attitude towards online clinical modules: a cross-sectional study among medical students from two countries

**DOI:** 10.12688/f1000research.130374.1

**Published:** 2023-07-04

**Authors:** Heraa Islam, Mohsin Nazeer Muhammed, Sindhura Lakshmi, Aditi Kapoor, Afraz Jahan, Akhila Doddamani, Nagaraja Kamath, Muhammed Ehsan, Suma Nair

**Affiliations:** 1 Kasturba Medical College, Manipal, Karnataka, 576104, India; 2 Department of Forensic Medicine, Kasturba Medical College, Manipal, Karnataka, 576104, India; 3 Department of Pathology, Kasturba Medical College, Manipal, Karnataka, 576104, India; 4 Department of Community Medicine, Kasturba Medical College, Manipal, Karnataka, 576104, India; 5 Department of Orthopaedics, KIMSHEALTH, Trivandrum, Kerala, 695029, India

**Keywords:** E-Learning, Perception, Assessment, COVID-19

## Abstract

The coronavirus disease (COVID-19) pandemic has significantly affected the world, including the education system, in various ways. In this study, we intended to explore the merits and demerits of online clinical learning and its effect on medical education from a student’s perspective. The study also assessed final-year medical students’ perception of and attitude towards, online clinical modules. This observational study was carried out in the Department of Community Medicine, Kasturba Medical College, Manipal (KMC) in collaboration with King’s College London, UK (KCL). In our study, a total of 42 students were enrolled, with 37 students from KMC and 5 students from KCL. In total 81% of students reported that they were not willing to continue with the online mode of learning. The abrupt switch to e-learning without prior preparation has exposed some pitfalls that must be attended to. Contrary to other fields, the medical field places much importance on offline clinical teaching, which has recently been impacted by the shift to online teaching. The survey responses were analysed for the improvisation of online clinical modules as well as to come up with better ideas and outcomes since this mode of learning may have to continue till the spread of the disease is under control.

## Introduction

The coronavirus disease (COVID-19) pandemic has significantly affected the world, including the education system, in various ways. The COVID-19 pandemic forced many schools and colleges to remain closed temporarily. In several parts of the world, many students missed at least one semester, with some students missing as much as a year of offline teaching. Teaching in medical school has traditionally been didactic, with clinical postings in the hospital. The WHO (World Health Organization) labeled the coronavirus outbreak a pandemic on March 11, 2020. ^1^^,^^2^ To battle this, several novel methods were employed like social distancing, masking, hand hygiene, and vaccination. The education sector has undergone substantial changes so that it can cope with the current situation. ^3^ Universities and colleges have employed a transition from traditional face-to-face teaching to online teaching or a hybrid of face-to-face teaching with online modules. ^4^ As the time spent on online education increases, a study in the United States showed that many educators are transforming their face-to-face teaching into an online format. ^5^ Therefore, to build a robust teaching tool to continue medical teaching during the pandemic, it is necessary to involve the students’ ideas and feedback to improve the online teaching modules. Online modules have restricted students from learning through bedside clinical teaching. However, this was necessary as there was a significant risk of medical students contracting the virus and spreading it within the community. ^6^^,^^7^ In this study we explored the merits and demerits of online clinical learning, its effect on medical education from the perspective of a student and assessed the perception and attitude of final year medical students towards online clinical modules.

## Methods

This observational study was carried out in the Department of Community Medicine, Kasturba Medical College (KMC) Manipal, and King’s College London (KCL), UK. In our study, a total of 42 students were enrolled from KCL (5 students) and KMC (37 students). The recruitment of participants was through Google forms, data from which were directly populated to the primary student researcher from KMC. Final year MBBS students from KCL and KMC college were RANDOMLY picked up or the survey. The authors from KMC analyzed the combined data.

### Inclusion criteria

Final year MBBS students from KCL (who participated in the Virtual Global Health Elective between the two institutions) and students from KMC Manipal who were in Final year MBBS, as of June 30th, 2020, and underwent clinical teaching on an online platform during their academic year.

### Exclusion criteria

Students attempting the final year exam for the second (or more) time were excluded from the study.

A questionnaire was used to conduct the survey. It was structured on an electronic database using Google forms and it was open for one month. The questionnaire was divided into four sections. The first section contained the disclaimer and the informed consent. This was followed by the participant information sheet in the second section. The third section addressed the general information on the participants like gender, which college they belong to, the year of medical school they are in, and the duration of online teaching exposure they have received. The final section of the questionnaire asks the students various questions about online clinical modules, their perception and attitude toward e-learning, and the applicability of online clinical teaching as a modus operandi of teaching in the future. The questionnaire provided to the participants was in English. The characteristics and replies of respondents were analyzed using descriptive statistics such as frequencies and percentages.

Informed consent was obtained from the participants before the study initiation. The study was approved (526/2021, dated July 14 ^th^, 2021) by the Institutional Ethics Committee (IEC, Registration No- ECR/146/Inst/KA/2013/RR-19 clearance) from KMC. Data obtained through this survey will not be transferred to the foreign collaborator. Collaboration with KCL was only in place until the collection of data through a Google form created by the primary student researcher. This data will be directly accessible only to the primary student researcher and will not be shared with the foreign collaborator.

## Results

**Figure 1.**
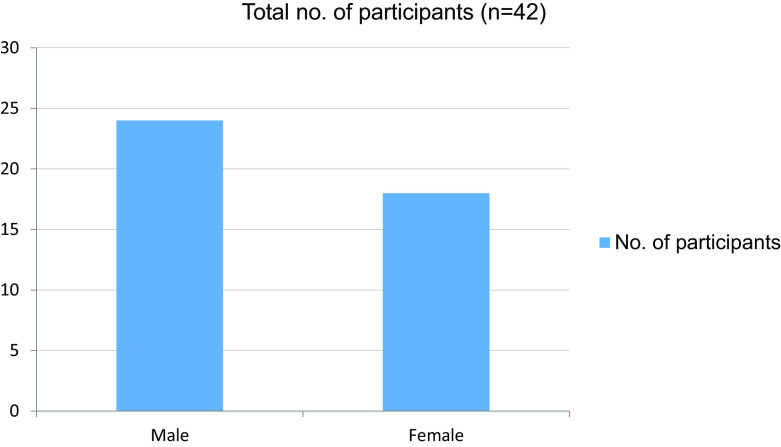
Gender wise distribution.

**Figure 2.**
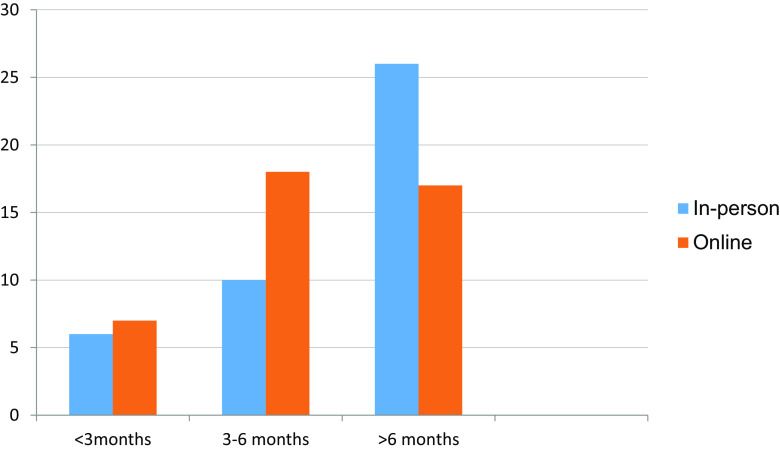
Duration of clinical exposure received in person and online.

**Figure 3.**
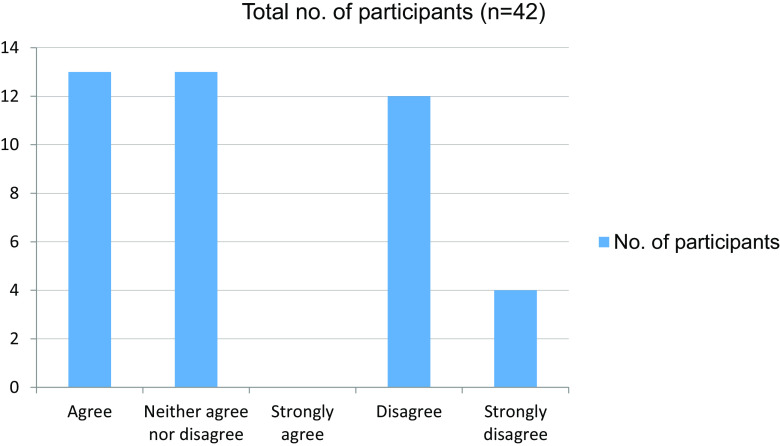
Able to grasp clinical concepts well through online sessions online.

**Figure 4.**
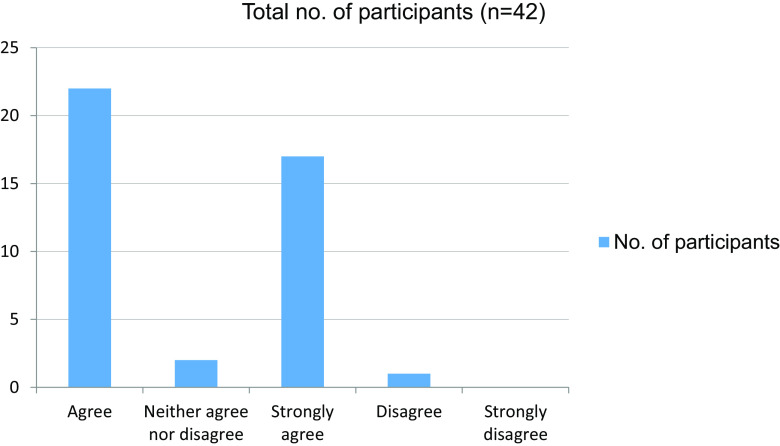
Attention affected in comparison with attending Clinics in person.

**Figure 5.**
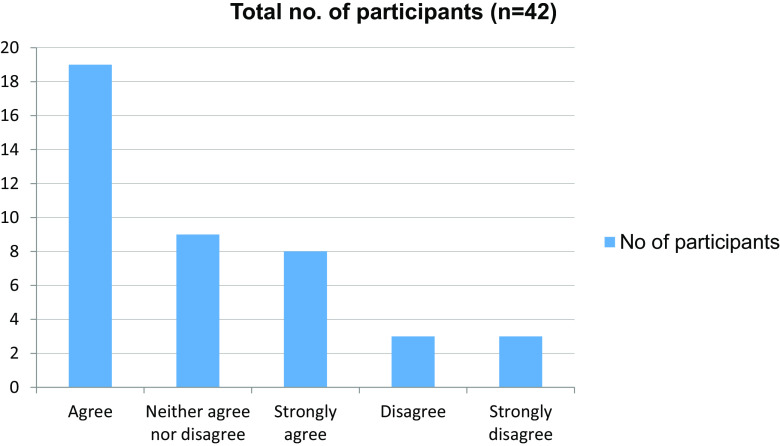
Change in motivation level in the absence of peers.

**Figure 6.**
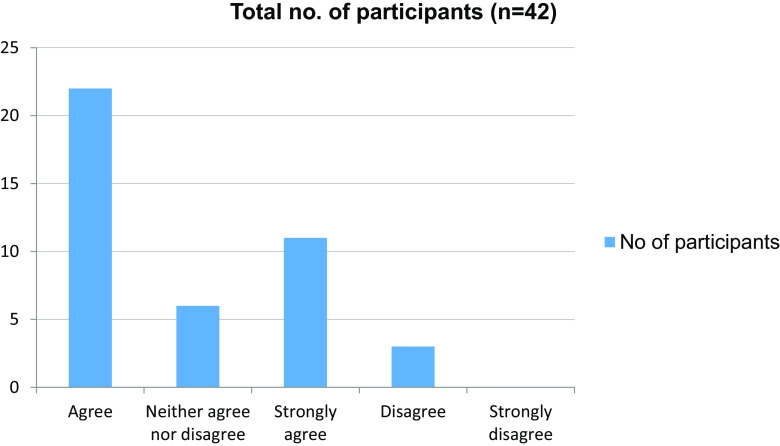
Learning atmosphere distractions.

**Figure 7.**
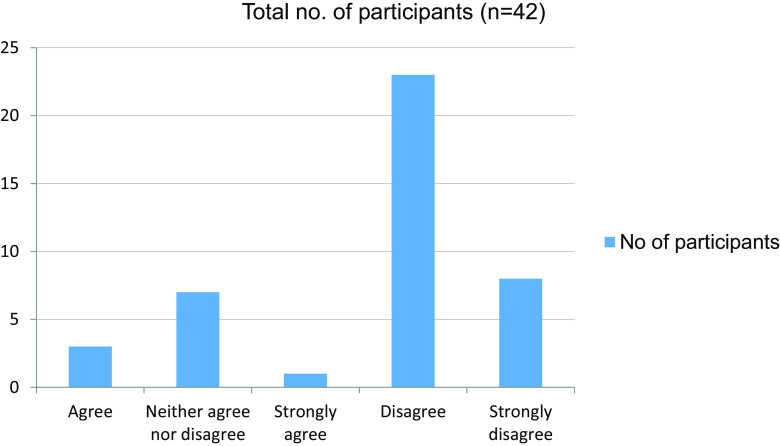
Trained by online classes to take clinical cases independently.

**Figure 8.**
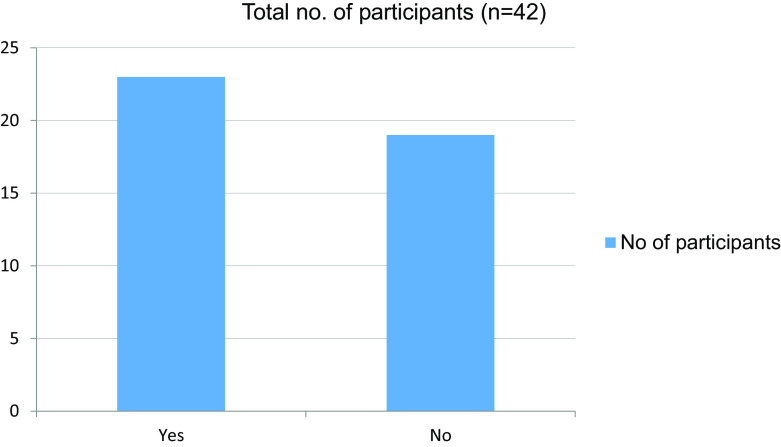
Interpersonal skills affected during the pandemic.

**Figure 9.**
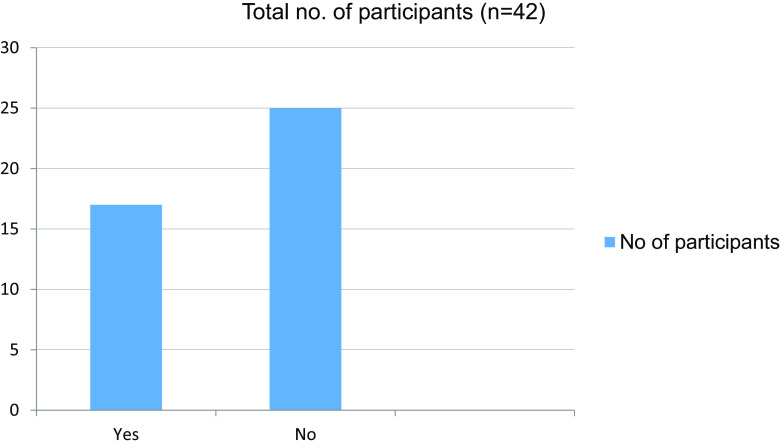
Giving online presentations during class is challenging.

**Figure 10.**
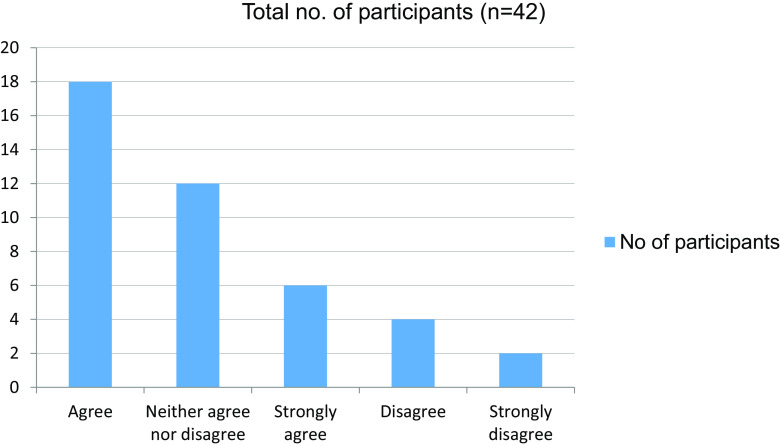
Able to get doubts cleared easily during online classes.

**Figure 11.**
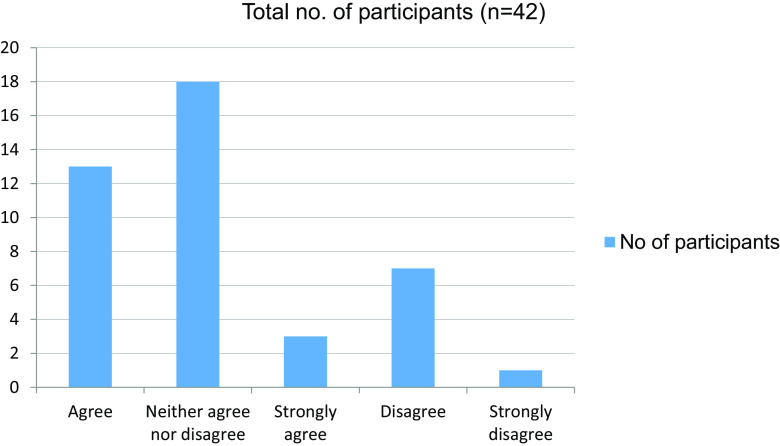
Demonstrating a clinical sign on a patient in the future would be challenging.

**Figure 12.**
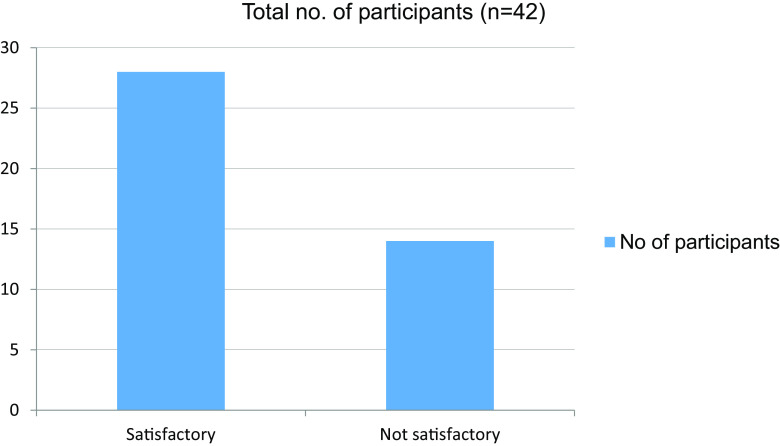
Performance in final clinical evaluation.

**Figure 13.**
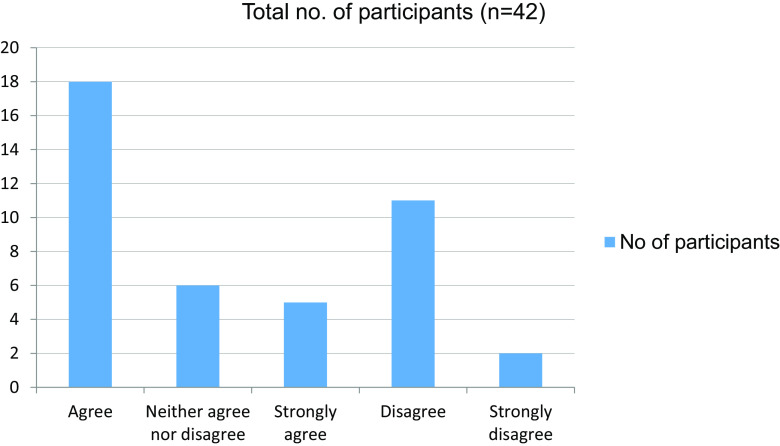
Familiar with hospital procedures like sending investigations and preparing discharge letters.

**Figure 14.**
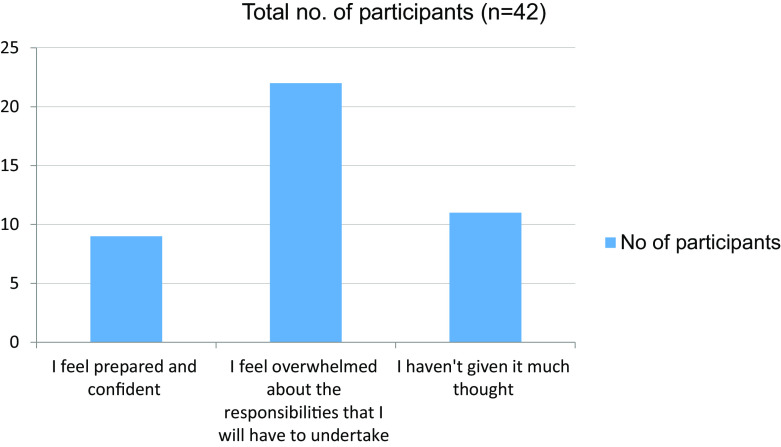
Working as a junior doctor in the following year.

**Figure 15.**
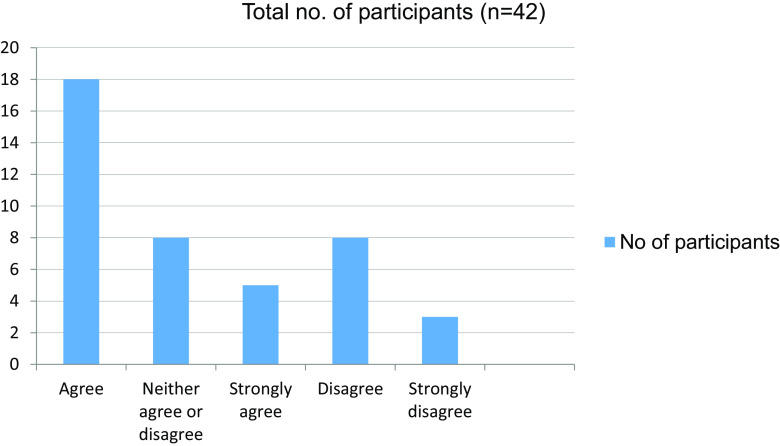
Online mode of learning has given more time to explore extracurricular interests.

**Figure 16.**
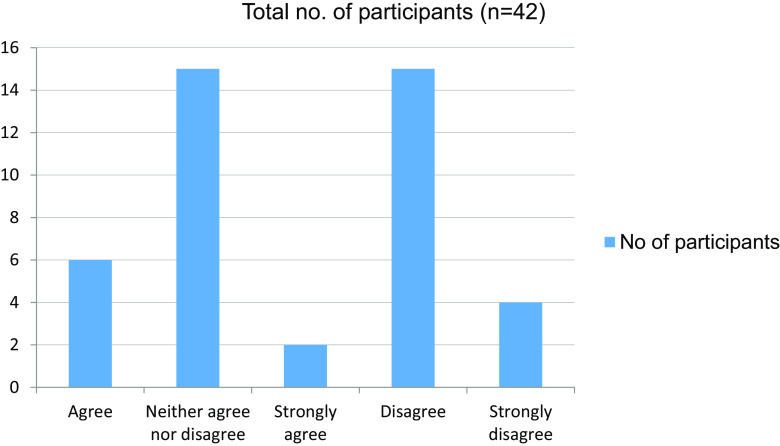
Online mode of learning is interesting.

**Figure 17.**
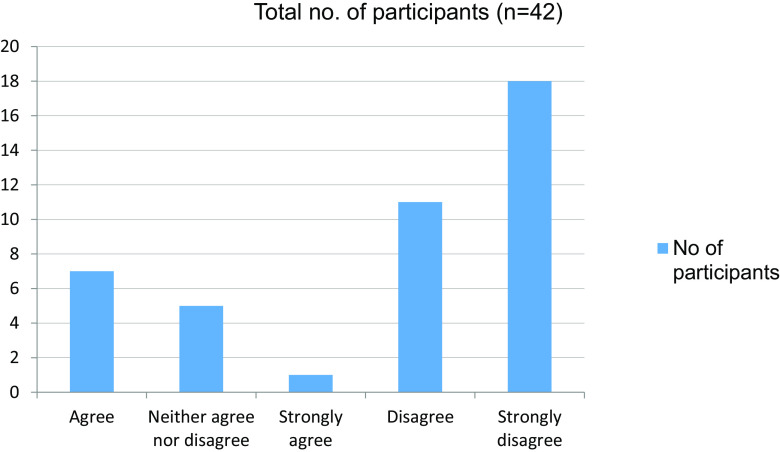
Wish to continue online mode of learning in the future.

In our study, a total of 42 students were enrolled, 24 were male and 18 were female ( Table 1 and Figure 1). ^20^ 26 out of 42 students received in-person clinical exposure for more than 6 months ( Table 2 and Figure 2). Around 16 students were not able to grasp clinical concepts well through online sessions ( Table 3 and Figure 3). In total, 39 students felt that their attention had been affected in online mode in comparison with attending clinics in person ( Table 4 and Figure 4). In the absence of peers, the motivation level was altered in 36 of our students ( Table 5 and Figure 5). Because of online clinical modules a lot of atmosphere distractions were present as per our study ( Table 6 and Figure 6). More than half the students disagreed to the fact that they were trained by online class to take clinical cases independently ( Table 7 and Figure 7). 28 of 42 students felt that their interpersonal skills were affected due to the pandemic ( Table 8 and Figure 8). Giving online presentation to the class was challenging to 17 out of 42 students ( Table 9 and Figure 9). At the same time most of the students were able to get their doubts solved during online lectures ( Table 10 and Figure 10). Due to online teaching, students also felt that demonstrating a clinical sign on a patient in future would be challenging ( Table 11 and Figure 11). 14 out of 42 students were not satisfied with their performance in final clinical evaluation ( Table 12 and Figure 12). More than half the students were familiar with hospital procedures like sending investigations and preparing discharge letters ( Table 13 and Figure 13). In addition, 22 students were overwhelmed about the responsibilities that they will have to undertake as a junior doctor ( Table 14 and Figure 14). More than half the students in the study agreed on the fact that online mode of learning has given them more time to explore extracurricular interests ( Table 15 and Figure 15). The online mode of learning was not interesting for students, and they did not wish to continue with this ( Tables 16, 17 and Figures 16, 17).

**Table 1.** Gender wise distribution.

Gender	No. of participants
Male	24
Female	18
Total	42

**Table 2.** Duration of clinical exposure received in person and online.

	In-person	Online
<3 months	6	7
3-6 months	10	18
>6 months	26	17
Total	42	42

**Table 3.** Able to grasp clinical concepts well through online sessions.

	No. of participants
Agree	13
Neither agree nor disagree	13
Strongly agree	0
Disagree	12
Strongly disagree	4
Total	42

**Table 4.** Attention has been affected in comparison with attending Clinics in person.

	No. of participants
Agree	22
Neither agree nor disagree	2
Strongly agree	17
Disagree	1
Strongly disagree	0
Total	42

**Table 5.** Change in motivation level evident in the absence of peers.

	No. of participants
Agree	19
Neither agree nor disagree	9
Strongly agree	8
Disagree	3
Strongly disagree	3
Total	42

**Table 6.** Learning atmosphere distractions are present.

	No. of participants
Agree	22
Neither agree nor disagree	6
Strongly agree	11
Disagree	3
Strongly disagree	0
Total	42

**Table 7.** Trained by online classes to take clinical cases independently.

	No. of participants
Agree	3
Neither agree nor disagree	7
Strongly agree	1
Disagree	23
Strongly disagree	8
Total	42

**Table 8.** Interpersonal skills affected during the pandemic.

	No. of participants
Yes	23
No	19
Total	42

**Table 9.** Giving online presentations during class is challenging.

	No. of participants
Yes	17
No	25
Total	42

**Table 10.** Able to get doubts cleared easily during online classes.

	No. of participants
Agree	18
Neither agree nor disagree	12
Strongly agree	6
Disagree	4
Strongly disagree	2
Total	42

**Table 11.** Demonstrating a clinical sign on a patient in the future would be challenging.

	No. of participants
Agree	13
Neither agree nor disagree	18
Strongly agree	3
Disagree	7
Strongly disagree	1
Total	42

**Table 12.** Performance in final clinical evaluation.

	No. of participants
Satisfactory	28
Not satisfactory	14
Total	42

**Table 13.** Familiar with hospital procedures like sending investigations and preparing discharge letters.

	No. of participants
Agree	18
Neither agree nor disagree	6
Strongly agree	5
Disagree	11
Strongly disagree	2
Total	42

**Table 14.** Working as a junior doctor in the following year.

	No. of participants
I feel prepared and confident	9
I feel overwhelmed about the responsibilities that I will have to undertake	22
I haven't given it much thought	11
Total	42

**Table 15.** Online mode of learning has given more time to explore extracurricular interests.

	No. of participants
Agree	18
Neither agree nor disagree	8
Strongly agree	5
Disagree	8
Strongly disagree	3
Total	42

**Table 16.** Online mode of learning is interesting.

	No. of participants
Agree	6
Neither agree nor disagree	15
Strongly agree	2
Disagree	15
Strongly disagree	4
Total	42

**Table 17.** Wish to continue online mode of learning in the future.

	No. of participants
Agree	7
Neither agree nor disagree	5
Strongly agree	1
Disagree	11
Strongly disagree	18
Total	42

## Discussion

During the COVID-19 pandemic, we investigated numerous components of e-learning that students at KMC Manipal and KCL encountered in terms of technical preparedness, teaching, and learning experience, engagement and communication, and assessment methodologies. While online learning may be more appropriate for preclinical students, it may not be the most ideal for their senior counterparts during the pandemic. The students from KCL experienced rotations like short- and long-term conditions, acute care, GP, and women’s health online while students from KMC Manipal had online rotations in medicine, surgery, OBGyn, orthopedics, and pediatrics. Importantly, these rotations are vital in imparting clinical knowledge to medical students. Moreover, it is crucial for final year students to have adequate contact with patients and bedside exposure to develop good communication and clinical skills.

Similarly, as an alternative to clinical placements, students at Imperial College London were exposed to Tele-teaching through computers in hospital settings. However, it was found that student-patient interaction was still lacking. ^8^

In our study, a total of 42 students were enrolled, 24 were male and 18 were female. Only four students from online classes felt that they were effectively trained to take clinical cases independently, while 37 students reported that they were not effectively trained to take clinical cases independently. Furthermore, 22 students agreed on learning atmosphere distractions and 19 students reported that their interpersonal skills were affected during the pandemic.

Every e-learning system establishes its foundation of computers, networks, communications, and technical facilities along with information technology professionals to continuously maintain and upgrade the system, train users, and provide technical support. ^9^ Appropriate technological support and maintenance of the available hardware and software are of great value for optimal utilization of technology by both educators and students alike. ^10^^–^^12^

E-learning modalities face several barriers and challenges. One of these is poor motivation and an expectation to be able to meet their personal and professional needs and goals. ^13^ Internal factors such as poor engagement, poor perception and motivation, high levels of anxiety and stress, and poor interactions between learners and facilitators all impede the process of learning and motivation. ^14^^,^^15^

The majority of the students reported disinterest in the online mode of learning. The students experienced fatigue from online classes and difficulty in enforcing self-discipline, along with the absence of usual social interaction which led to a decrease in attendance as well as engagement during the virtual sessions. Stress has been more often associated with e-learning than with traditional learning. ^16^^,^^17^

In our study, only 19% of students wish to continue the online mode of learning while 81% of students reported that they were not willing to continue with the online format. Varied results have been reported in the literature when comparing student satisfaction in face-to-face and online courses, with a higher proportion of students preferring face-to-face classes. ^18^

There were diverse opinions obtained from our survey regarding the experience of students with the technical aspects of online classes. Some felt that these classes helped them explore their extracurricular interests and, as the need of the hour, the classes were good and manageable. However, most students were not satisfied, found it difficult to focus, and felt that it should never be a substitute for the offline practical learning experience. Some of the technical drawbacks included network and connectivity issues, difficulties with the application, and video/audio quality. These issues made it difficult, especially when the students were looking forward to a topic of interest. Moreover, while some facilitators were more adept at using online platforms than others, oftentimes the time taken to set up the online lecture took away from the overall classroom experience. The same professors who had previously engaged the classroom and involved the students during face-to-face lectures had now resorted to monotonous online lectures. Thereby, most of the students participating in this study stated that they would not like to take more e-learning classes if they were given the option. The consensus was that the long and draining online classes had now given the students renewed vigor and zeal to attend face-to-face classes whenever possible.

## Conclusion

The sudden shift to e-learning without prior preparedness has revealed some pitfalls that need to be addressed. The central hypothesis is that COVID-19 has impacted the academic performance and clinical skills of medical students. The responses were analyzed for improvisation of online clinical modules as well as to come up with better ideas and outcomes since this mode of learning may have to continue till the spread of the disease is under control. In the near future, with the re-emergence of the pandemic and the advent of modernization and the digital world, e-learning and e-teaching should be incorporated in the medical curriculum along with clinical teaching.

### Recommendations

Medical education places an emphasis on offline clinical teaching with the most ideal learning known to happen at the bedside. As a result, given the current pandemic scenario, medical educators must develop new approaches to imparting knowledge to medical students; they can no longer rely on markers and whiteboards, or chalks and blackboards.

Since the online mode of learning may have to continue for the foreseeable future, one of the effective methods that can be adopted to create the most effective multimedia learning experiences are Mayer’s 12 principles of multimedia learning. ^19^ These principles can be used as guidelines to develop productive digital learning experiences.

## Data Availability

Figshare: Perception and attitude towards online clinical modules: A Cross-sectional study among medical students from 2 countries, https://doi.org/10.6084/m9.figshare.21857076.v2. ^20^ This project contains the following underlying data:
• Survey (Responses).xlsx Data are available under the terms of the Creative Commons Zero “No rights reserved” data waiver (CC0 1.0 Public domain dedication).
