# Perception and attitude towards online clinical modules: a cross-sectional study among medical students from two countries

**DOI:** 10.12688/f1000research.130374.2

**Published:** 2024-01-15

**Authors:** Heraa Islam, Mohsin Nazeer Muhammed, Sindhura Lakshmi, Aditi Kapoor, Afraz Jahan, Akhila Doddamani, Nagaraja Kamath, Muhammed Ehsan, Suma Nair

**Affiliations:** 1 Kasturba Medical College, Manipal, Karnataka, 576104, India; 2 Department of Forensic Medicine, Kasturba Medical College, Manipal, Karnataka, 576104, India; 3 Department of Pathology, Kasturba Medical College, Manipal, Karnataka, 576104, India; 4 Department of Community Medicine, Kasturba Medical College, Manipal, Karnataka, 576104, India; 5 Department of Orthopaedics, KIMSHEALTH, Trivandrum, Kerala, 695029, India

**Keywords:** E-Learning, Perception, Assessment, COVID-19

## Abstract

COVID-19 has deeply affected the world in various aspects including the education system worldwide. In this study, we intended to explore the merits and demerits of online clinical learning and its effect on medical education from a student’s perspective. The study also assessed the perception and attitude of final-year medical students towards online clinical modules. This observational study was carried out in the Department of Community Medicine, Kasturba Medical College, Manipal (KMC) in collaboration with King’s College London, UK (KCL). In our study, a total of 42 students were enrolled, with 37 students from KMC and 5 students from KCL. 81% of students reported that they were not willing to continue the online mode of learning. The abrupt switch to e-learning without prior preparedness has exposed some pitfalls that must be attended to. Contrary to other fields, the medical field places high importance on offline clinical teaching which has recently been impacted by online teaching. The survey responses were analyzed for improvisation of online clinical modules as well as to come up with better ideas and outcomes since this mode of learning may have to continue till the spread of the disease is under control.

## Introduction

The coronavirus disease (COVID-19) pandemic has significantly affected the world, including the education system, in various ways. The COVID-19 pandemic forced many schools and colleges to remain closed temporarily. In several parts of the world, many students missed at least one semester, with some students missing as much as a year of offline teaching. Teaching in medical school has traditionally been didactic, with clinical postings in the hospital. The WHO (World Health Organization) labeled the coronavirus outbreak a pandemic on March 11, 2020. ^1^^,^^2^ To battle this, several novel methods were employed like social distancing, masking, hand hygiene, and vaccination. The education sector has undergone substantial changes so that it can cope with the current situation. ^3^ Universities and colleges have employed a transition from traditional face-to-face teaching to online teaching or a hybrid of face-to-face teaching with online modules. ^4^ As the time spent on online education increases, a study in the United States showed that many educators are transforming their face-to-face teaching into an online format. ^5^ Therefore, to build a robust teaching tool to continue medical teaching during the pandemic, it is necessary to involve the students’ ideas and feedback to improve the online teaching modules. Online modules have restricted students from learning through bedside clinical teaching. However, this was necessary as there was a significant risk of medical students contracting the virus and spreading it within the community. ^6^^,^^7^ In this study we explored the merits and demerits of online clinical learning, its effect on medical education from the perspective of a student and assessed the perception and attitude of final year medical students towards online clinical modules.

When compared to more traditional, in-person learning environments, there is some evidence of student anxiety regarding online learning. There is also data regarding how the media portrays emergency circumstances. In the event of a disease outbreak, learning may be both facilitated and impeded by public agencies, political bodies, and research institutes). ^8^ Furthermore, crisis management is an ongoing process in which public institutions and entities may find it challenging to apply “lessons learned” or prior knowledge from disaster scenarios in a developing emergency scenario. ^9^ Although there is a lot of room for student participation using online learning tools, this may not be the same as in-person or on-campus learning. ^10^ According to survey results, fewer than half of adults questioned said that an online course was equivalent to one completed in a classroom, and there seemed to be a higher risk of plagiarism when learning online. ^11^ When compared to traditional in-person instruction, distance learning that is exclusively provided by videoconferencing can also result in inferior academic grades and lower course satisfaction. ^12^ Furthermore, there are real obstacles to overcome in the context of online learning, such as the "learning curve" towards active learning and computer confidence that affects both students and teachers. ^13^ Nevertheless, other research has discovered that student performance (as determined by exam results) might be comparable when the same professor teaches both in-person and online classes. ^14^

## Methods

**Study design –** Observational study.

**Study setting –** Department of Community Medicine, Kasturba Medical College (KMC), Manipal, and King’s College London (KCL), UK.

**Study participants –** Total of 42 students were enrolled from KCL (5 students) and KMC (37 students). The recruitment of participants was through Google forms, data from which were directly populated to the primary student researcher from KMC. Final year MBBS students from KCL and KMC college were RANDOMLY picked up or the survey.

### Inclusion criteria

Final year MBBS students from KCL (who participated in the Virtual Global Health Elective between the two institutions) and students from KMC Manipal who were in Final year MBBS, as of June 30th, 2020, and underwent clinical teaching on an online platform during their academic year.

### Exclusion criteria

Students attempting the final year exam for the second (or more) time were excluded from the study.

### Data collection procedure

Informed consent was obtained from the participants before the study initiation. Questionnaire was used to conduct the survey. It was structured on an electronic database using Google forms and it was open for one month. The questionnaire was divided into four sections. The first section contained the disclaimer and the informed consent. This was followed by the participant information sheet in the second section. The third section addressed the general information on the participants like gender, which college they belong to, the year of medical school they are in, and the duration of online teaching exposure they have received. The final section of the questionnaire asks the students various questions about online clinical modules (prepared as per NMC Competency Based Undergraduate Curriculum), their perception and attitude toward e-learning, and the applicability of online clinical teaching as a modus operandi of teaching in the future. The questionnaire provided to the participants was in English.

### Statistical method

The characteristics and replies of respondents were analyzed using descriptive statistics such as frequencies and percentages. Data obtained through this survey will not be transferred to the foreign collaborator. Collaboration with KCL was only in place until the collection of data through a Google form created by the primary student researcher. This data will be directly accessible only to the primary student researcher and will not be shared with the foreign collaborator.

### Ethical approval

The study was approved (526/2021, dated July 14 ^th^, 2021) by the Institutional Ethics Committee (IEC, Registration No-ECR/146/Inst/KA/2013/RR-19 clearance) from KMC.

## Results

**Figure 1.**
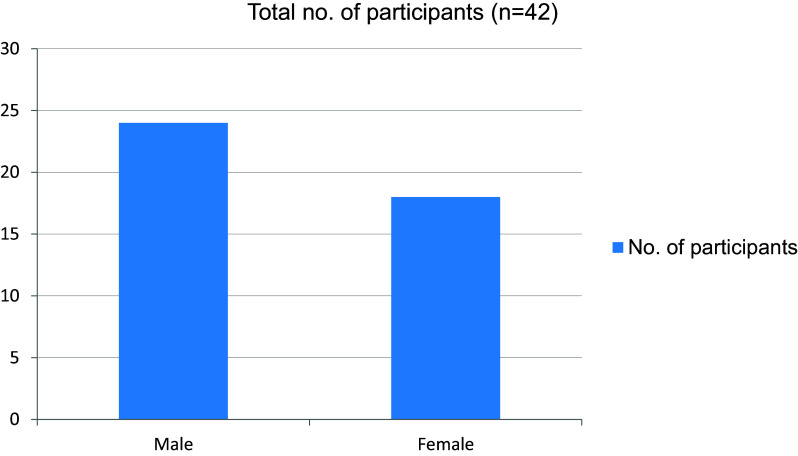
Gender wise distribution.

**Figure 2.**
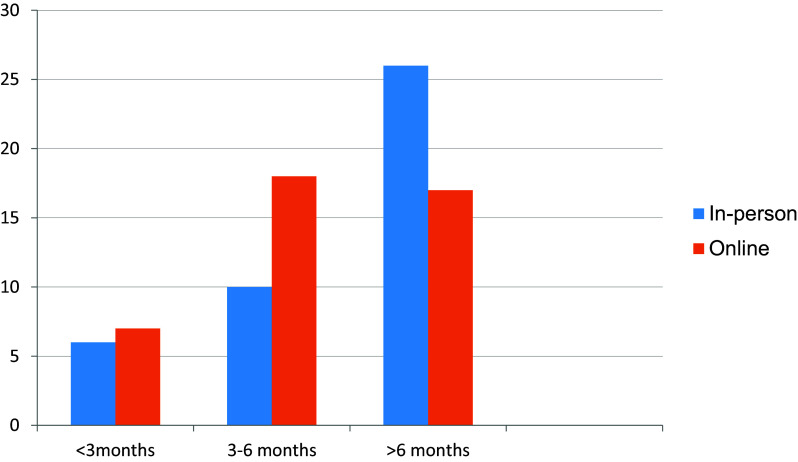
Duration of clinical exposure received in person and online.

**Figure 3.**
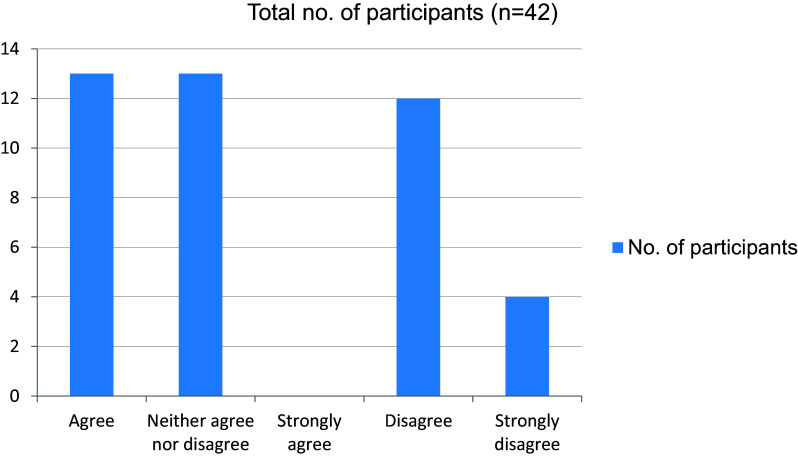
Able to grasp clinical concepts well through online sessions online.

**Figure 4.**
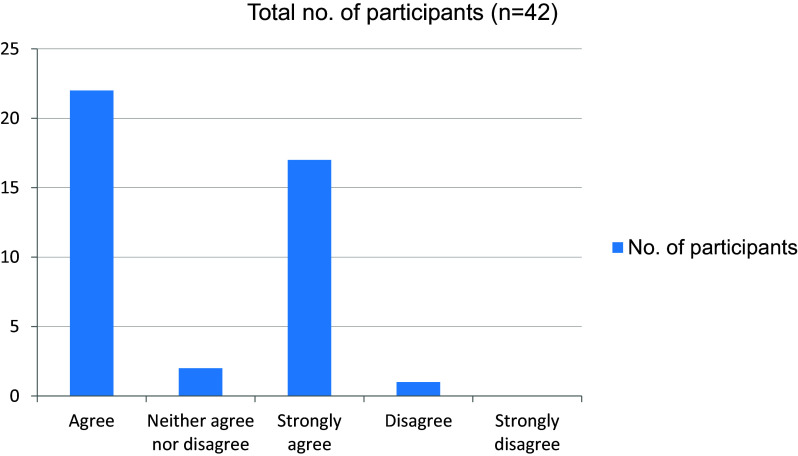
Attention affected in comparison with attending Clinics in person.

**Figure 5.**
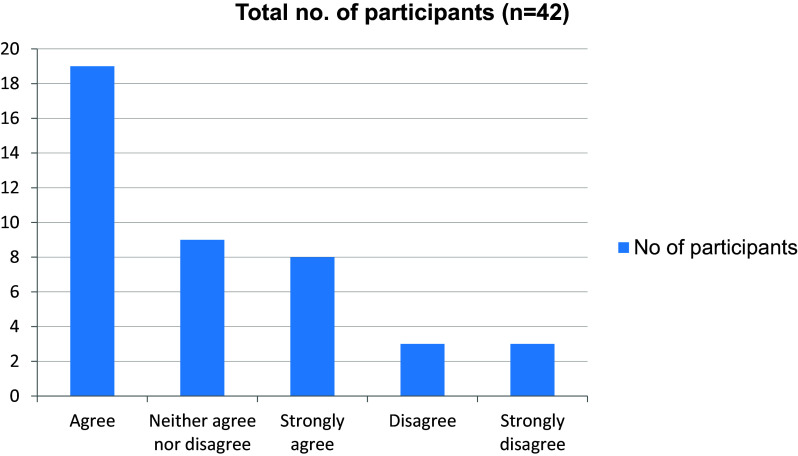
Change in motivation level in the absence of peers.

**Figure 6.**
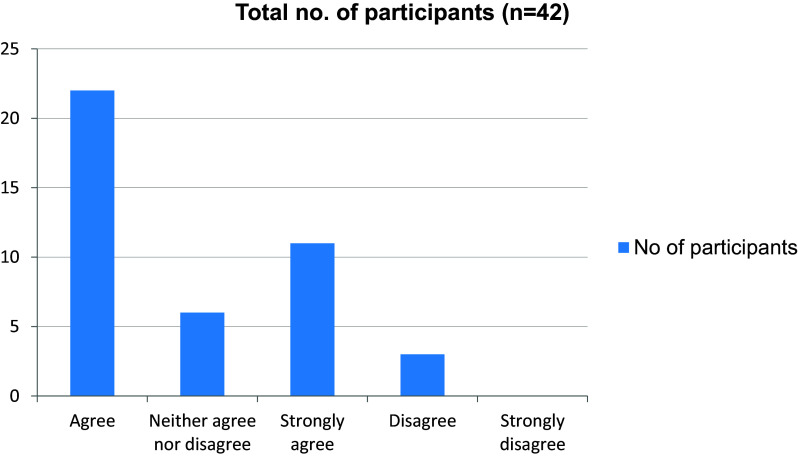
Learning atmosphere distractions.

**Figure 7.**
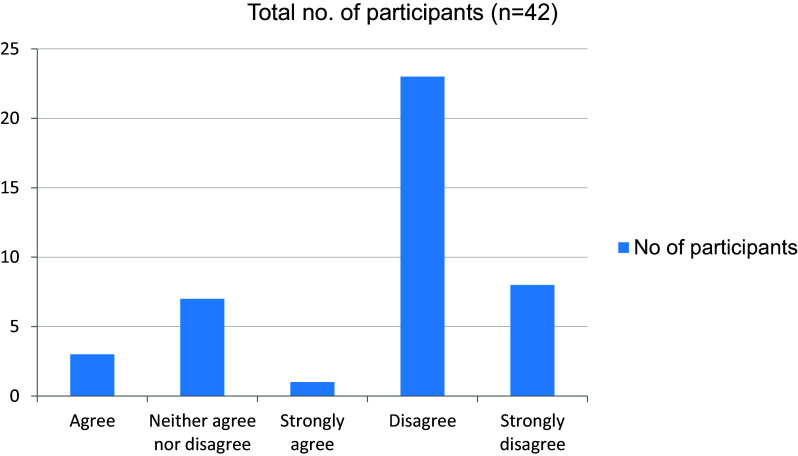
Trained by online classes to take clinical cases independently.

**Figure 8.**
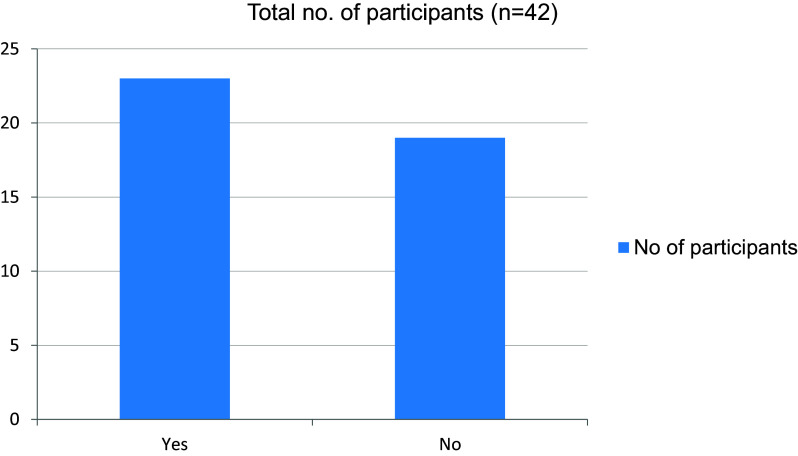
Interpersonal skills affected during the pandemic.

**Figure 9.**
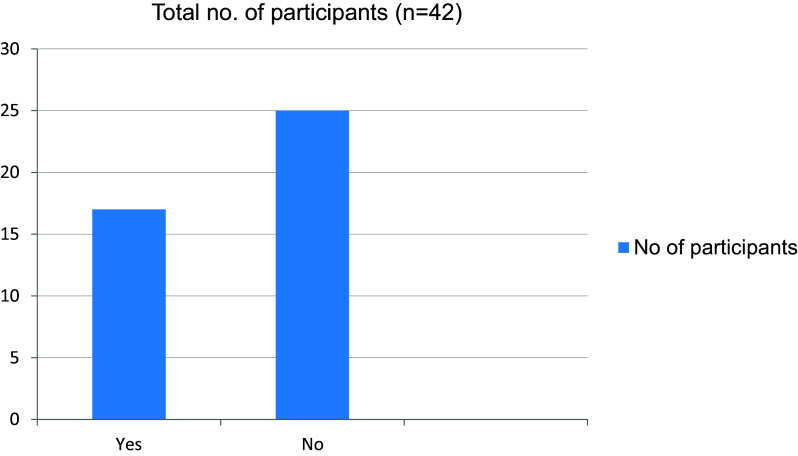
Giving online presentations during class is challenging.

**Figure 10.**
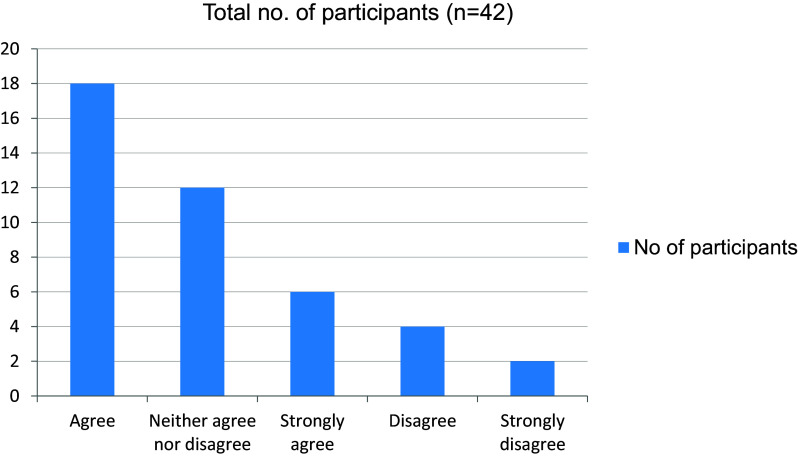
Able to get doubts cleared easily during online classes.

**Figure 11.**
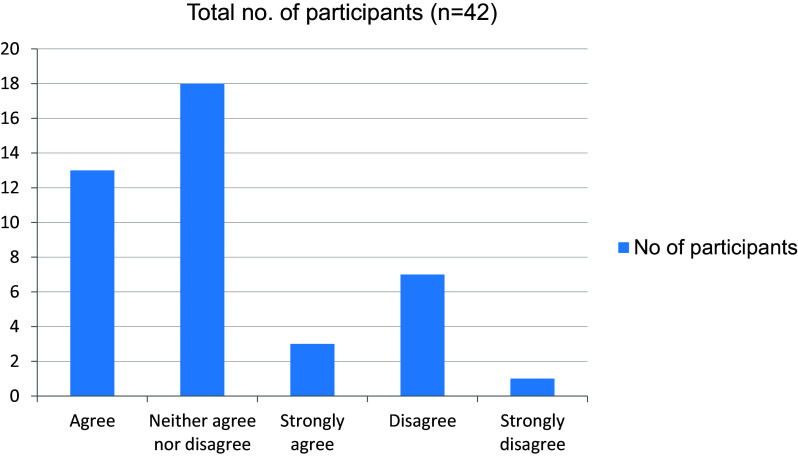
Demonstrating a clinical sign on a patient in the future would be challenging.

**Figure 12.**
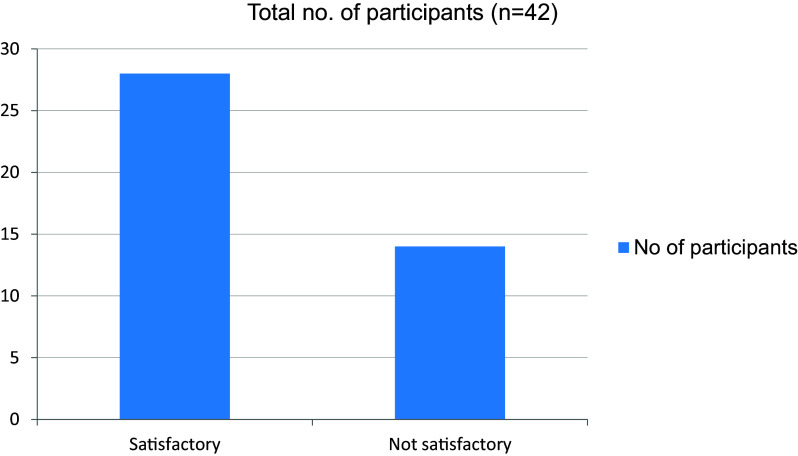
Performance in final clinical evaluation.

**Figure 13.**
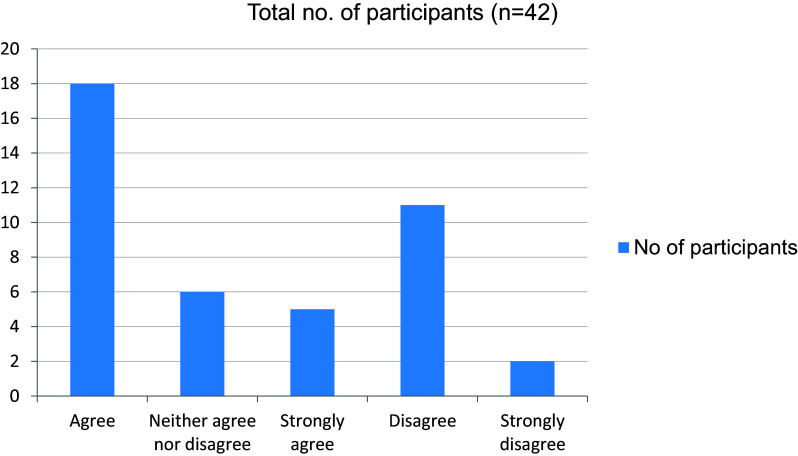
Familiar with hospital procedures like sending investigations and preparing discharge letters.

**Figure 14.**
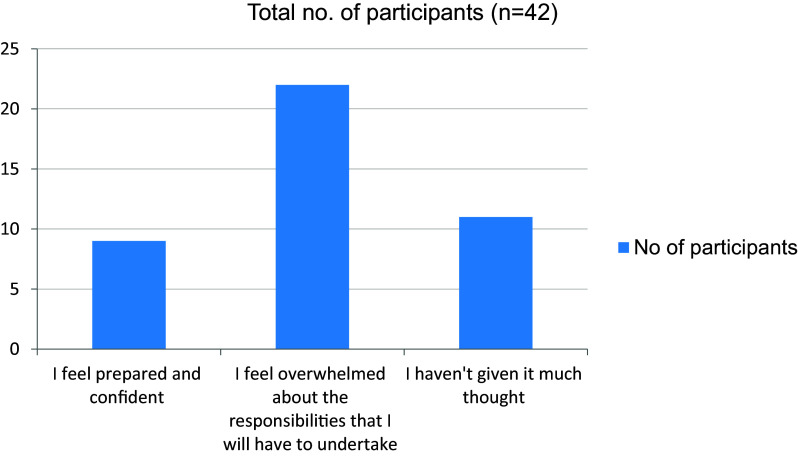
Working as a junior doctor in the following year.

**Figure 15.**
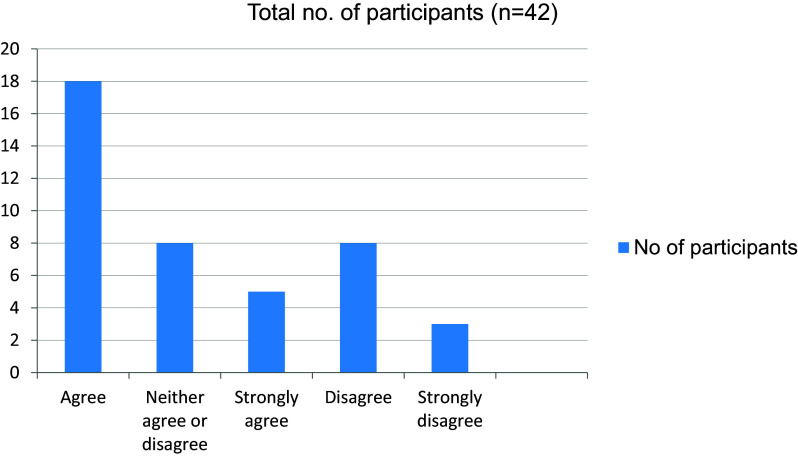
Online mode of learning has given more time to explore extracurricular interests.

**Figure 16.**
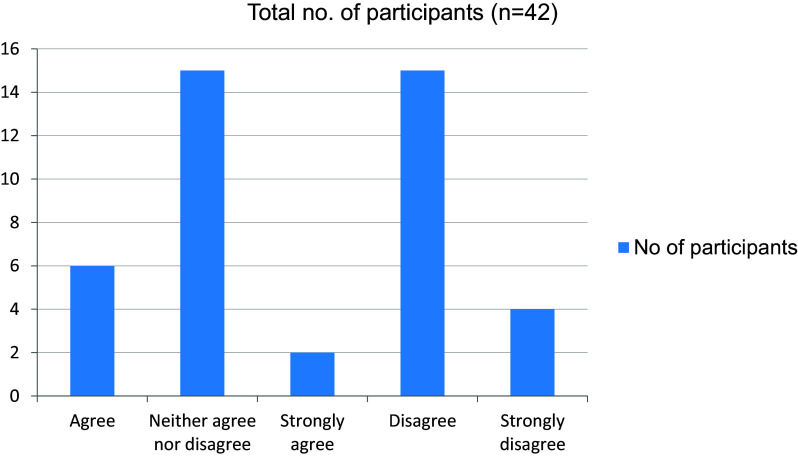
Online mode of learning is interesting.

**Figure 17.**
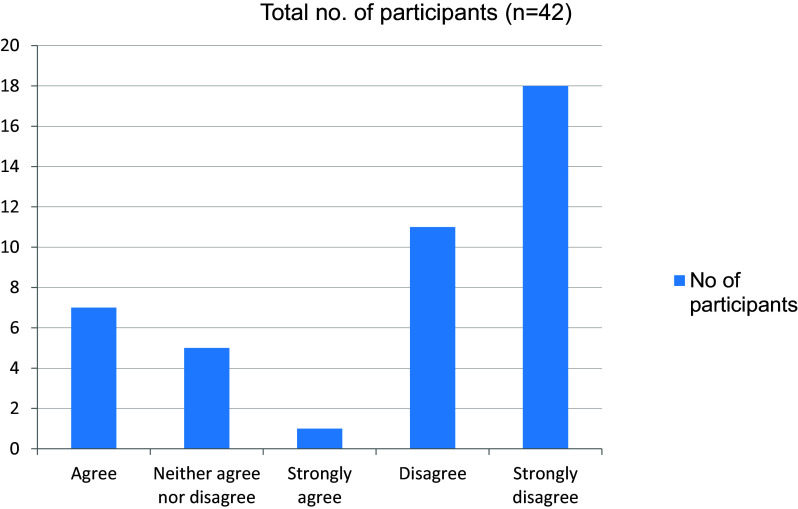
Wish to continue online mode of learning in the future.

In our study, a total of 42 students were enrolled, 24 were male and 18 were female ( Figure 1). ^20^ 26 out of 42 students received in-person clinical exposure for more than 6 months ( Figure 2). Around 16 students were not able to grasp clinical concepts well through online sessions ( Figure 3). In total, 39 students felt that their attention had been affected in online mode in comparison with attending clinics in person ( Figure 4). In the absence of peers, the motivation level was altered in 36 of our students ( Figure 5). Because of online clinical modules a lot of atmosphere distractions were present as per our study ( Figure 6). More than half the students disagreed to the fact that they were trained by online class to take clinical cases independently ( Figure 7). 28 of 42 students felt that their interpersonal skills were affected due to the pandemic ( Figure 8). Giving online presentation to the class was a difficult task to 17 out of 42 students ( Figure 9). At the same time most of the students were able to get their doubts solved during online lectures ( Figure 10). Due to online teaching, students also felt that demonstrating a clinical sign on a patient in future would be challenging ( Figure 11). 14 out of 42 students were not satisfied with their performance in final clinical evaluation is measured on clinical case scenario evaluation individually ( Figure 12). More than half the students were familiar with hospital procedures like sending investigations and preparing discharge letters ( Figure 13). In addition, 22 students were overwhelmed about the responsibilities that they will have to undertake as a junior doctor ( Figure 14). More than half the students in the study agreed on the fact that online mode of learning has given them more time to explore extracurricular interests ( Figure 15). The online mode of learning was not interesting for students, and they did not wish to continue with this ( Figures 16, 17). The technical difficulties faced by the students during the study were poor internet connectivity, Compatibility issues and Limited interaction.

## Discussion

We looked into many aspects of e-learning that students at KMC Manipal and KCL encountered during the COVID-19 epidemic, including technical readiness, the teaching and learning process, engagement and communication, and assessment techniques. Online instruction might not be the best option for senior colleagues during the epidemic, even though it might be more suitable for preclinical students. While students from KMC Manipal had online rotations in medicine, surgery, OBGyn, orthopedics, and pediatrics, KCL students experienced rotations in short- and long-term conditions, acute care, general practice, and women's health. Significantly, these rotations are essential for teaching medical students clinical expertise. Additionally, in order for final-year students to develop effective communication skills, they must have enough bedside exposure and patient interaction and clinical skills.

Similarly, students at Imperial College London were exposed to tele-teaching via computers in hospital settings as an alternative to clinical rotations. It was discovered that there was still a paucity of student-patient engagement. ^15^ There were 24 male students and 18 female students involved in our study. Of the students who took online classes, only four thought they had received enough training to be able to handle clinical cases on their own, while 37 said they had not received enough training. Additionally, 19 students stated that the pandemic had an impact on their interpersonal skills, and 22 students felt that there were distractions in the studying environment. Every e-learning system starts with a base of computers, networks, communications, and technical facilities, and information technology specialists keep these systems updated and maintained on a constant basis to upgrade the system, train users, and provide technical support. ^16^ Appropriate technological support and maintenance of the available hardware and software are of great value for optimal utilization of technology by both educators and students alike. ^17^^–^^19^

E-learning approaches encounter various obstacles and difficulties. Poor motivation and the expectation that one can achieve one's own needs and ambitions, both personally and professionally, are two of these. ^20^ Thirteen The process of learning and motivation is hampered by internal variables include low engagement, low motivation and perception, excessive levels of stress and anxiety, and bad relationships between facilitators and learners. ^21^^,^^22^ The vast majority of pupils expressed a lack of interest in learning online.

Due to the lack of regular social connection, exhaustion from online learning, and difficulties imposing self-discipline, the students' attendance and involvement during the virtual sessions decreased. E-learning has been linked to stress more frequently than traditional learning. ^23^^,^^24^

Only 19% of students in our study said they would like to continue learning in an online format, whilst 81% said they would not be willing to continue in an online style. When comparing student satisfaction in in-person versus online courses, varying outcomes have been documented in the literature; a greater percentage of students seem to favor in-person instruction. ^25^

According to a recently published study from King Abdulaziz University in Jeddah, Saudi Arabia, medical students accepted online learning during the pandemic lockdowns to a reasonable extent. Approximately half of the study's respondents said online learning was superior to or comparable to in-person instruction. ^26^

Our study yielded a variety of responses about students' experiences with the technological aspects of online learning. According to some, these classes allowed them to pursue their extracurricular interests, and they were appropriate and manageable given the current circumstances. Nonetheless, the majority of students expressed dissatisfaction, difficulty focusing, and belief that technology should never replace the in-person, hands-on learning experience. A few of the technical problems were with the application, the network and connectivity, and the audio and video quality. It was challenging because of these problems, particularly when the kids were anticipating a topic of interest. Furthermore, even while some facilitators were more skilled at using online resources than others, the time it took to set up the online lecture frequently detracted from the overall learning environment. The same instructors who had previously involved the students and the classroom in in-person lectures had suddenly turned to dry online lectures. As a result, the majority of the study's participants said that, given the choice, they would not prefer to enroll in any further online courses. Everyone agreed that after taking lengthy and taxing online classes, the students now had more energy and enthusiasm to attend in-person classes whenever possible.

## Conclusion

The sudden shift to e-learning without prior preparedness has revealed some pitfalls that need to be addressed. The central hypothesis is that COVID-19 has impacted the academic performance and clinical skills of medical students. The responses were analyzed for improvisation of online clinical modules as well as to come up with better ideas and outcomes since this mode of learning may have to continue till the spread of the disease is under control. In the near future, with the re-emergence of the pandemic and the advent of modernization and the digital world, e-learning and e-teaching should be incorporated in the medical curriculum along with clinical teaching.

### Recommendations

Medical education places an emphasis on offline clinical teaching with the most ideal learning known to happen at the bedside. As a result, given the current pandemic scenario, medical educators must develop new approaches to imparting knowledge to medical students; they can no longer rely on markers and whiteboards, or chalks and blackboards.

Since the online mode of learning may have to continue for the foreseeable future, one of the effective methods that can be adopted to create the most effective multimedia learning experiences are Mayer’s 12 principles of multimedia learning. ^27^ These principles can be used as guidelines to develop productive digital learning experiences.

## Data Availability

Figshare: Perception and attitude towards online clinical modules: A Cross-sectional study among medical students from 2 countries, https://doi.org/10.6084/m9.figshare.21857076.v2. ^27^ This project contains the following underlying data:
• Survey (Responses).xlsx Data are available under the terms of the Creative Commons Zero “No rights reserved” data waiver (CC0 1.0 Public domain dedication).
