# Supplementary material for: Perception and attitude towards online clinical modules: a cross-sectional study among medical students from two countries
Source: F1000Res. 2024 Jan 15;12:776. Originally published 2023 Jul 4. [Version 2] doi: 10.12688/f1000research.130374.2 (PMC10904952; doi:10.12688/f1000research.130374.2)
Supplement: Peer review history

# Reviewer report: Reviewer response for version 2

**DOI:** 10.5256/f1000research.158201.r237851

**Published:** 2024-02-11

**Authors:** Ivan Buljan

**Affiliations:** 1 Faculty of Humanities and Social Sciences in Split, Split, Croatia

Thank you for the invitation. The authors responded to my queries, and I have nothing to add.

Is the work clearly and accurately presented and does it cite the current literature?

No

If applicable, is the statistical analysis and its interpretation appropriate?

Partly

Are all the source data underlying the results available to ensure full reproducibility?

Yes

Is the study design appropriate and is the work technically sound?

Yes

Are the conclusions drawn adequately supported by the results?

Yes

Are sufficient details of methods and analysis provided to allow replication by others?

No

Reviewer Expertise:

Evidence based medicine education, Science communication, Psychology

I confirm that I have read this submission and believe that I have an appropriate level of expertise to confirm that it is of an acceptable scientific standard.

# Reviewer report: Reviewer response for version 2

**DOI:** 10.5256/f1000research.158201.r237852

**Published:** 2024-01-22

**Authors:** Gehanath Baral

**Affiliations:** 1 Nepal Health Research Council, Kathmandu, Nepal; 2 Nobel Medical College, Kathmandu University, Dhulikhel, Central Development Region, Nepal

Revisions made by the authors are now acceptable.

Is the work clearly and accurately presented and does it cite the current literature?

Partly

If applicable, is the statistical analysis and its interpretation appropriate?

Partly

Are all the source data underlying the results available to ensure full reproducibility?

No

Is the study design appropriate and is the work technically sound?

No

Are the conclusions drawn adequately supported by the results?

No

Are sufficient details of methods and analysis provided to allow replication by others?

Partly

Reviewer Expertise:

Obsterics, Gynecology, Oncology, Clinical Research

I confirm that I have read this submission and believe that I have an appropriate level of expertise to confirm that it is of an acceptable scientific standard.

# Reviewer report: Reviewer response for version 1

**DOI:** 10.5256/f1000research.143129.r193973

**Published:** 2023-10-04

**Authors:** Ivan Buljan

**Affiliations:** 1 Faculty of Humanities and Social Sciences in Split, Split, Croatia

Thank you for the opportunity to review this manuscript. 

 I commend the authors for exploiting COVID situation to assess the attitudes of students towards online learning. However, in my opinion, the reporting needs to be further expanded, including relevant literature sources, reporting guidelines and more straightforward statistical reporting.

 My comments are the following:

 a) In the background section more references are needed, especially scoping reviews of the literature. Further expansion of background in several paragraphs would also be helpful (especially is those would answer the questions "What is this about and why it is important?", What have previous studies discovered, Which questions remained unanswered, What did we decide to do?).

 b) Methods section should be more itemized. The recommendation is to use STROBE checklist for observational studies. Here I would emphasize on the importance of sample size calculation, since it seems that the sample of 42 students can be understood as relatively small.

 c) The results section can be shortened, since the analysis is mostly descriptive, and I would recommend that all results are entered in a single table, rather than series of small tables. Moreover, Likert type scales can be entered as medians and interquartile ranges, rather than frequencies. Also, all percentages in the description of the sample should be removed and presented as simple frequencies, because there are less than 100 participants in the sample. 

 d) Finally, the discussion should rely on the evidence granted from the Results, which it does. The recommendation is to restructure it, by putting the most important results first, and providing more precise explanations for findings, with literature sources.

 Once again, thank you for the opportunity to review this manuscript.

Is the work clearly and accurately presented and does it cite the current literature?

No

If applicable, is the statistical analysis and its interpretation appropriate?

Partly

Are all the source data underlying the results available to ensure full reproducibility?

Yes

Is the study design appropriate and is the work technically sound?

Yes

Are the conclusions drawn adequately supported by the results?

Yes

Are sufficient details of methods and analysis provided to allow replication by others?

No

Reviewer Expertise:

Evidence based medicine education, Science communication, Psychology

I confirm that I have read this submission and believe that I have an appropriate level of expertise to state that I do not consider it to be of an acceptable scientific standard, for reasons outlined above.

# Response

**Published:** 2023-11-20

**Authors:** Mohsin Naseer Muhammed

**Affiliations:** Kasturba Medical College Hospital, India

1. In the background section more references are needed, especially scoping reviews of the literature. Further expansion of background in several paragraphs would also be helpful (especially is those would answer the questions "What is this about and why it is important?", What have previous studies discovered, Which questions remained unanswered, What did we decide to do?).

**Response:** Further paragraphs and references are added in background section as per the suggestion.

 2. Methods section should be more itemized. The recommendation is to use STROBE checklist for observational studies. Here I would emphasize on the importance of sample size calculation, since it seems that the sample of 42 students can be understood as relatively small.

**Response:** Method section was itemized and modified accordingly.  

 3. The results section can be shortened, since the analysis is mostly descriptive, and I would recommend that all results are entered in a single table, rather than series of small tables. Moreover, Likert type scales can be entered as medians and interquartile ranges, rather than frequencies. Also, all percentages in the description of the sample should be removed and presented as simple frequencies, because there are less than 100 participants in the sample.

**Response:** The result section was shortened and corrected as per the suggestions.

 4. Finally, the discussion should rely on the evidence granted from the Results, which it does. The recommendation is to restructure it, by putting the most important results first, and providing

 more precise explanations for findings, with literature sources.

**Response:** Discussion section was edited and restructured providing the relevant evidence first.

# Reviewer report: Reviewer response for version 1

**DOI:** 10.5256/f1000research.143129.r198153

**Published:** 2023-10-04

**Authors:** Carlos Trenado

**Affiliations:** 1 Heinrich Heine University, Duesseldorf, Germany

The present observational study focuses on differences between online and offline learning in the medical setting. The authors designed a questionnaire in which students expressed their views. The study is valuable in highlighting some issues with the sudden adoption of an educational style, nevertheless some issues remain.

 Comments:

 - The authors expressed that participants were exposed to online clinical training, was online training similar to all students at the same institution? Was it different? Please provide more information about the online training received and any differences between them. Was the online lecture presented with slides? Are there any other features such as sound, etc.?

 - What was the duration of a typical online session?

 - While the authors emphasize several elements as contributing factors for a low of interest in online education, it is not clear which one of them was more influential. I strongly recommend a regression analysis to extract information about what factors were more influential in predicting a particular preference.

 - Please provide data on students that suffered from technical difficulties. I think this factor should be considered in the regression model.

 - I recommend the use of bar graphs instead of tables for each of the items described. Please omit tables.

Is the work clearly and accurately presented and does it cite the current literature?

Partly

If applicable, is the statistical analysis and its interpretation appropriate?

No

Are all the source data underlying the results available to ensure full reproducibility?

Partly

Is the study design appropriate and is the work technically sound?

Partly

Are the conclusions drawn adequately supported by the results?

No

Are sufficient details of methods and analysis provided to allow replication by others?

Partly

Reviewer Expertise:

Neuroscience, Neuroeducation, Clinical Neuroscience. Computational Modeling

I confirm that I have read this submission and believe that I have an appropriate level of expertise to confirm that it is of an acceptable scientific standard, however I have significant reservations, as outlined above.

# Response

**Published:** 2023-11-17

**Authors:** Mohsin Naseer Muhammed

**Affiliations:** Kasturba Medical College Hospital, India

Comment 1.

 The authors expressed that participants were exposed to online clinical training, was online training similar to all students at the same institution? Was it different? Please provide more information about the online training received and any differences between them. Was the online lecture presented with slides? Are there any other features such as sound, etc.?

 Response: The online training given in our study was same for all the students in both the institutions.

 Information regarding online training modules were added. The online lecture was presented with slides in Zoom and Google platforms which includes clinical contents, videos, images, diagrams and flow charts etc.

 Comment 2.

 What was the duration of a typical online session?

 Response: One hour for one topic

 Comment 3.

 While the authors emphasize several elements as contributing factors for a low of interest in online education, it is not clear which one of them was more influential. I strongly recommend a regression analysis to extract information about what factors were more influential in predicting a particular preference.

 Response: Our study utilized a descriptive quantitative design to obtain the opinions of the respondents.

 The recommendation by the reviewer for regression analysis will be followed to extract information about the factors involved to predict particular preference in our future studies.

 Comment 4.

 Please provide data on students that suffered from technical difficulties. I think this factor should be considered in the regression model.

 Response: The technical difficulties faced by the students during the study was added.

 Comment 5.

 I recommend the use of bar graphs instead of tables for each of the items described. Please omit tables.

 Response: Tables were deleted as per the recommendation

# Reviewer report: Reviewer response for version 1

**DOI:** 10.5256/f1000research.143129.r198158

**Published:** 2023-10-04

**Authors:** Gehanath Baral

**Affiliations:** 1 Nepal Health Research Council, Kathmandu, Nepal; 2 Nobel Medical College, Kathmandu University, Dhulikhel, Central Development Region, Nepal

1. Write the interpretation of information in a Table or Figure as a summary finding followed by a Table or Figure for easy understanding of the result. At the end of the paragraph, there must be a reference to the relevant Table or Figure in parentheses.
2. Keep either Table or Figure for the same information. Information is duplicated.
3. Table/Figure 1 information on the gender of participants can be displayed in text only. There is unnecessary space occupied by these.
4. Table/Figure 7 information on the “Trained by online classes to take clinical cases independently” should have been verified to publish the result. It means that research should not highlight misperceptions if any. Verifiable information should be presented.
5. Table/Figure 9 information on “Giving online presentations during class is challenging” doesn’t clarify the presentations delivered by teachers or students to judge in terms of a CHALLENGE.
6. Table/Figure 12 information on “Performance in final clinical evaluation” is not clear whether it is on patient evaluation or clinical reports or case scenarios; and how the performance was measured or gauged.
7. Table/Figure 13. Familiar with hospital procedures like sending investigations and preparing discharge letters needs objective verification. Such learning requires a set/validated teaching procedure – is it used or not?
8. Table/Figure 14 information on “Working as a junior doctor in the following year” seems to be an assumption or hypothetical. For this research, design should be different like time series information on online and offline experiences.
9. Table/Figure 17. Wish to continue online mode of learning in the future. Without looking at its impact, the data shown here will not be realistic. Because the level of knowledge and skill acquisition by a medical student is for real-time implementation, theoretical knowledge will not suffice for medical education.
10. Discussion on technology is not supported by results.

Is the work clearly and accurately presented and does it cite the current literature?

Partly

If applicable, is the statistical analysis and its interpretation appropriate?

Partly

Are all the source data underlying the results available to ensure full reproducibility?

No

Is the study design appropriate and is the work technically sound?

No

Are the conclusions drawn adequately supported by the results?

No

Are sufficient details of methods and analysis provided to allow replication by others?

Partly

Reviewer Expertise:

Obsterics, Gynecology, Oncology, Clinical Research

I confirm that I have read this submission and believe that I have an appropriate level of expertise to state that I do not consider it to be of an acceptable scientific standard, for reasons outlined above.

# Response

**Published:** 2023-11-20

**Authors:** Mohsin Naseer Muhammed

**Affiliations:** Kasturba Medical College Hospital, India

1. Write the interpretation of information in a Table or Figure as a summary finding followed by a Table or Figure for easy understanding of the result. At the end of the paragraph, there must be a reference to the relevant Table or Figure in parentheses.

**Response:** Interpretation of information in a Table or Figure as a summary finding was added.

 2. Keep either Table or Figure for the same information. Information is duplicated

**Response:** Bar graphs was retained and Tables were deleted as per the recommendation

 3. Table/Figure 1 information on the gender of participants can be displayed in text only. There is unnecessary space occupied by these.

**Response: **Table was deleted

 4. Table/Figure 7 information on the “Trained by online classes to take clinical cases independently” should have been verified to publish the result. It means that research should not highlight misperceptions if any. Verifiable information should be presented.

**Response:** The question was verified with the students before analysing the result part.

 5. Table/Figure 9 information on “Giving online presentations during class is challenging” doesn’t clarify the presentations delivered by teachers or students to judge in terms of a CHALLENGE.

**Response:** The term challenging was changed to difficult task.         

 6. Table/Figure 12 information on “Performance in final clinical evaluation” is not clear whether it is on patient evaluation or clinical reports or case scenarios; and how the performance was measured or gauged.

**Response:** The Performance in final clinical evaluation is measured on clinical case scenario evaluation individually and it was added now.

 7. Table/Figure 13. Familiar with hospital procedures like sending investigations and preparing discharge letters needs objective verification. Such learning requires a set/validated teaching procedure – is it used or not?

**Response:** Such learning requires a set/validated teaching procedure was used for students to familiarize with hospital procedures like sending investigations and preparing discharge letters

 8. Table/Figure 14 information on “Working as a junior doctor in the following year” seems to be an assumption or hypothetical. For this research, design should be different like time series information on online and offline experiences.

**Response: **Though the question was hypothetical, since it was a pandemic and necessity of doctors were highly required at the time of study. Hence it was added to know their mind set.

 9. Table/Figure 17. Wish to continue online mode of learning in the future. Without looking at its impact, the data shown here will not be realistic. Because the level of knowledge and skill acquisition by a medical student is for real-time implementation, theoretical knowledge will not suffice for medical education.

**Response:** Majority of the medical students strongly disagreed online mode of learning in the future since hands on skills will not be adequate. Hence it was added in result to highlight its importance among medical students.

 10. Discussion on technology is not supported by results.

**Response:** Discussion part is modified accordingly.
